# Effect of Acupuncture on Neuroplasticity of Stroke Patients with Motor Dysfunction: A Meta-Analysis of fMRI Studies

**DOI:** 10.1155/2021/8841720

**Published:** 2021-06-02

**Authors:** Qiuyi Lv, Guixing Xu, Yuxin Pan, Tongtong Liu, Xiaodong Liu, Linqing Miao, Xing Chen, Lan Jiang, Jie Chen, Yingjia He, Rong Zhang, Yihuai Zou

**Affiliations:** ^1^Department of Neurology and Stroke Center, Dongzhimen Hospital, The First Affiliated Hospital of Beijing University of Chinese Medicine, Beijing, China; ^2^The Acupuncture and Tuina School/The 3rd Teaching Hospital, Chengdu University of Traditional Chinese Medicine, Chengdu, China; ^3^Institute of Neuroscience, Chinese Academy of Sciences, Shanghai, China; ^4^Guang'anmen Hospital, China Academy of Chinese Medical Sciences, Beijing, China; ^5^Beijing University of Chinese Medicine, Beijing, China; ^6^Beijing Institute of Technology, Beijing, China; ^7^School of Life Science, Peking University, Beijing, China

## Abstract

**Objective:**

To analyze the pattern of intrinsic brain activity variability that is altered by acupuncture compared with conventional treatment in stroke patients with motor dysfunction, thus providing the mechanism of stroke treatment by acupuncture.

**Methods:**

Chinese and English articles published up to May 2020 were searched in the PubMed, Web of Science, EMBASE, and Cochrane Library databases, China National Knowledge Infrastructure, Chongqing VIP, and Wanfang Database. We only included randomized controlled trials (RCTs) using resting-state fMRI to observe the effect of acupuncture on stroke patients with motor dysfunction. R software was used to analyze the continuous variables, and Seed-based d Mapping with Permutation of Subject Images (SDM-PSI) was used to perform an analysis of fMRI data. *Findings*. A total of 7 studies comprising 143 patients in the treatment group and 138 in the control group were included in the meta-analysis. The results suggest that acupuncture treatment helps the healing process of motor dysfunction in stroke patients and exhibits hyperactivation in the bilateral basal ganglia and insula and hypoactivation in motor-related areas (especially bilateral BA6 and left BA4).

**Conclusion:**

Acupuncture plays a role in promoting neuroplasticity in subcortical regions that are commonly affected by stroke and cortical motor areas that may compensate for motor deficits, which may provide a possible mechanism underlying the therapeutic effect of acupuncture.

## 1. Introduction

Stroke is a syndrome characterized by an acute onset of neurologic deficit resulted from ischemia or hemorrhage. It became the leading cause of death and years of life lost (YLLs) at the national level in China [1]. Patients with stroke are susceptible to several complications, including pain, dysphagia, pneumonias, incontinence, depression, and somatic dyskinesia, which usually impede clinical recovery and require specific intervention [2, 3]. In this review, we mainly focus on the motor dysfunction after stroke. As a common and severe complication of stroke, motor dysfunction recovery requires multidisciplinary treatment and still remains a clinical challenge [4]. Seeking an effective and safe alternative therapy meets the requirement of the Healthy China 2030 plan [5].

Acupuncture, a key modality of Traditional Chinese Medicine (TCM) therapy, is recommended by the World Health Organization (WHO) as an alternative and complementary strategy for poststroke treatment [6, 7]. Acupuncture has been widely used to relieve pain and improve motor or neurological function [8, 9]. Sufficient previous studies including animal experiments, clinical trials, and systematic reviews have proven that acupuncture was beneficial for stroke rehabilitation [10]. Although the application of acupuncture has shown good clinical efficacy, the mechanisms of acupuncture remain unknown. One hypothesis suggests that acupuncture may induce neuroplasticity to exert therapeutic effects [11–13].

Neuroplasticity refers to the capability of the nervous system to modify and reorganize the brain structure and function in response to impairment or experience; various behavioral, neurophysiological, and neuroimaging studies can document changes in the nervous system [14, 15]. One of the recommended modalities used to measure neuroplasticity in humans is resting-state functional magnetic resonance imaging (rs-fMRI), which is often used to assess resting-state networks in participants [16, 17]. The main analysis approaches of rs-fMRI are regional homogeneity (ReHo) and amplitude of low-frequency fluctuations (ALFFs). The former evaluates the temporal homogeneity of the regional blood oxygen level-dependent (BOLD) signal, and the latter reflects intrinsic brain low frequency [18, 19]. Aimed at a better understanding of the cerebral characters of acupuncture treatment, more and more acupuncture studies have applied rs-fMRI in the clinical trials. However, no consensus has been reached, possibly due to the small sample size or distinctions of experimental design between studies. For example, Schaechter et al. [20] have reported that acupuncture would activate the ipsilateral lesional motor cortex to improve motor function in patients with hemiparetic stroke, while others suggested that the basal ganglia would be predominantly activated by acupuncture [21].

Therefore, in order to elucidate which brain area plays a crucial role in the therapeutic effect of acupuncture, this review synthesized corresponding studies and introduced coordinate-based meta-analysis (CBMA) to quantitatively integrate the results of individual neuroimaging studies [22, 23]. The feature of CBMA is the use of effect sizes, which allows combination of peak coordinates with statistical parametric maps and it has been fully validated in many studies [24–28]. In the present study, we applied the Seed-based d Mapping with Permutation of Subject Images (SDM-PSI), which is a new generation algorithm for CBMA meta-analysis, to analyze the rs-fMRI data of included studies [29, 30]. The results would determine whether acupuncture has a differential effect on brain activity as compared to regular or conventional treatment. The apprehension of neuroplasticity mechanisms induced by acupuncture can not only elucidate the central mechanism of acupuncture but also possibly provide novel ideas for applying acupuncture to the treatment of other neurodegenerative diseases.

## 2. Methods

We conducted the meta-analysis in line with the *Cochrane Handbook for Systematic Reviews of Interventions*. All procedure followed the Preferred Reporting Items for Systematic Reviews and Meta-Analyses guidelines (PRISMA guidelines). The study was registered in the PROSPERO International prospective register of systematic reviews (registration number: CRD 42020185421).

### 2.1. Literature Search

Studies that examined the neuroprotective effect of acupuncture in stroke patients were included in the present study. The PubMed, EMBASE, and Cochrane Library databases, Web of Science, China National Knowledge Infrastructure (CNKI), Chongqing VIP (VIP), and the Wanfang Database (WF) were searched from inception until May 2020 by two independent researchers. The following English search terms were used: (stroke OR Poststroke OR Cerebrovascular Accident OR Cerebrovascular Apoplexy OR Apoplexy OR Brain Vascular Accident OR Cerebrovasc∗ OR brain∗ OR brain vasc∗ OR hemipleg∗ OR apoplex∗ OR CVA OR TIA) AND (acupuncture OR acupuncture therapy OR acupuncture treatment OR electroacupuncture OR electro-acupuncture OR acupuncture, ear OR ear acupuncture OR auriculotherapy OR scalp acupuncture) AND (RCT OR randomized controlled trial OR controlled clinical trial OR randomized OR clinical trial OR randomly OR RCT OR trial) AND (fMRI OR functional MRI OR functional magnetic resonance imaging OR neuroimaging). The languages of the trials were restricted to English or Chinese, and the search strategy for each database was based on its own unique characteristics.

### 2.2. Inclusion/Exclusion Criteria

Two researchers independently conducted the search and screened the titles, abstracts, and full texts of the papers. Studies were included based on the following criteria: (1) an RCT conducted in patients with stroke sequelae at any-stroke stage, of any age and gender; (2) manual acupuncture or electroacupuncture with or without other therapies in the treatment group, while other therapies including conventional rehabilitation or sham acupuncture in the control group; (3) involved whole-brain functional imaging (ReHo or ALFFs) in resting state; (4) three-dimensional coordinates (*x*, *y*, *z*) reported in standard stereotactic space Talairach or Montreal Neurological Institute (MNI); (5) if one study involved two or more comparable datasets, all samples were included; and (6) used secondary outcomes to assess clinical efficacy. The exclusion criteria were as follows: (1) the study only used region of interest (ROI) method; (2) the sample size in each group was less than 5; (3) if separate papers used the same or similar datasets, only the largest sample was included.

### 2.3. Data Extraction and Quality Assessment

Data were extracted from the included studies into a standard form with respect to publishing year, name of the first author, sample size, participants' condition, intervention design, fMRI method, and secondary outcome measures. Missing or unclear data for the meta-analysis were obtained after corresponding with the original authors by email or telephone. Two independent researchers carried out the data extraction; any disagreements were resolved by a third reviewer. This information is showed in Table 1. Quality assessment was based on Cochrane Risk of Bias Tool and carried out by two researchers independently. All the reports were evaluated according to the following seven criteria: random sequence generation, allocation concealment, blinding of participants and personnel, blinding of outcome assessment, incomplete outcome data, selective reporting, and other sources of bias. For each criterion, studies were judged to be at low, high, or unclear risk of bias.

### 2.4. Data Synthesis and Meta-Analysis

ReHo results: the differences of brain activity between the treatment group and the control group were analyzed by SDM-PSI (version 6.21, https://www.sdmproject.com/). A step-by-step tutorial is provided on this website. First, reported peak coordinates and effect sizes (*t* value, or equivalently from *z*-scores or *P* values) were extracted to prepare the peaks' text files. In the step of preprocessing, software will use those files to recreate the lower and upper bounds of the possible effect-size values of the studies. Second, the meta-analysis consisted in calculating the random-effects mean of the ReHo values and the mean map was weighted by the sample size and variance of each study. We reported results using family-wise error (FWE) correction (*P* < 0.05 and voxel extent ≥ 10) with the threshold-free cluster enhancement approach (TFCE) and 5000 permutations. The heterogeneity analyses were also assessed. The peak MNI coordinate would be extracted to derive standard heterogeneity statistics *I*^2^, and an *I*^2^ < 50% indicates low heterogeneity. Funnel plots were not performed because the amount of included studies (*n* = 7) was less than 10, but the Egger test was used to assess the publication bias. Finally, a metaregression analysis was carried out to figure out the relationship between ReHo changes and clinical variables. All results would be reported using the TFCE-based FWE corrected threshold (*P* < 0.05 and voxel extent ≥ 10).

Clinical variables: statistical analyses of continuous data were performed with R software, version 3.6.2 (R Foundation for Statistical Computing, Vienna, Austria), using the Meta and Metafor meta-analysis packages. Weighted mean difference (WMD) with 95% CIs was used. Heterogeneity was tested by the *I*^2^ statistic. We selected fixed or random-effects model to pool the data depending on whether the trials had good homogeneity (*P* > 0.10, *I*^2^ < 50%) or not. A forest plot was adopted to show the hypothesis test results, and if necessary, a sensitivity analysis or subgroup analysis would be performed to find sources of heterogeneity.

## 3. Results

### 3.1. Included Studies

**Figure 1:**
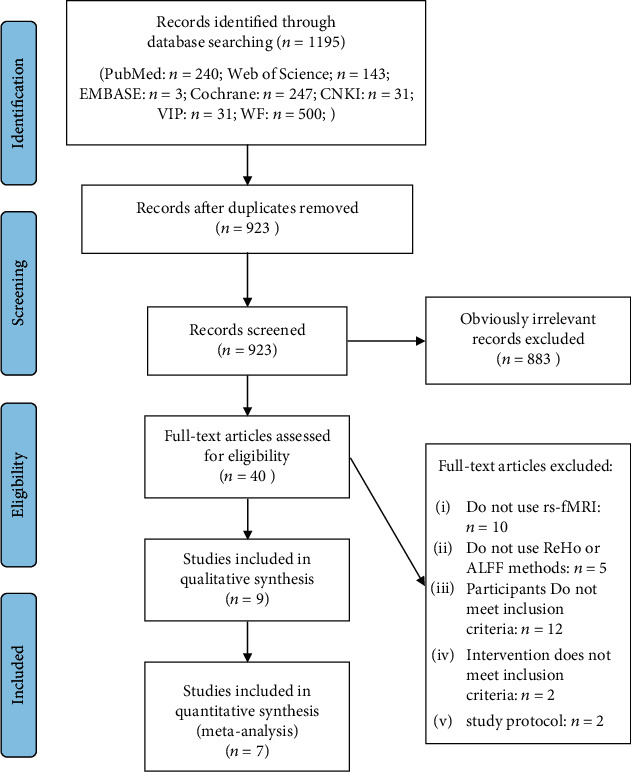
Flowchart for study inclusion and exclusion process.

The database search yielded 1195 articles. 272 duplicates were removed, and 883 articles were excluded after screening titles and abstracted. Of the 40 potentially relevant reports, 6 articles [31–36] and 7 studies (Chen et al. [35] conducted two trials at the same time) proved eligible after full-text screening (Figure 1).

Trials were published after 2000, and each study enrolled 10 to 100 patients (140 individuals in the treatment group and 138 in the control group). All studied recruited ischemic stroke patients. There were no significant differences in demographic baseline characteristics, including age, gender, disease status including symptom duration, or secondary outcome measures between the two groups. Three studies reported the patient limb dysfunction of the right side and the most lesions of brain regions had the left laterality (especially in the left basal ganglia) [32, 35, 36]. All patients in both groups received conventional treatment and, on this basis, participants in the treatment group underwent two or four courses (weeks) of manual acupuncture therapy, one study combined with sham acupuncture as comparison [32]. The manual acupuncture methods include lifting yang to dredging du meridian needling method (LYDDM) and Xingnao Kaiqiao needling method (XNKQ) and empirical acupoints. The LYDDM and XNKQ needling methods are based on long-term clinical experiments and were developed in by Professor Peilin Zhang and Xuemin Shi, respectively. And the application of empirical acupoints means that doctors manipulate the acupuncture based on their clinical experience. All studies reported rs-fMRI data by using ReHo variations to compare differences before and after treatment in patients from both groups. Clinical efficacy was examined by Fugl-Meyer assessment (FMA; *n* = 7), nervous functional deficiency scale (NDS; *n* = 3), Barthel index (BI; *n* = 4), or modified Barthel index (MBI; *n* = 3). The details and features of the studies are shown in Table 1.

### 3.2. Quality Assessment

**Figure 2:**
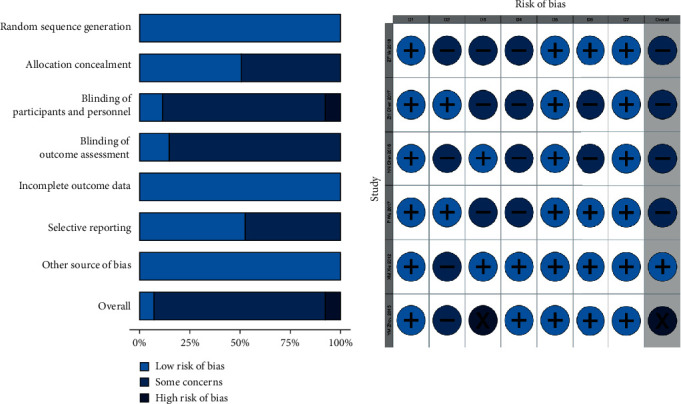
Risk of bias summary. Each color represents a level of risk of bias: light blue, low risk of bias; medium blue, unclear risk of bias; dark blue, high risk of bias. D1: random sequence generation; D2: allocation concealment; D3: blinding of participants and personnel; D4: blinding of outcome data; D5: incomplete outcome data; D6: selective reporting; D7: other sources of bias.

The quality assessment was based on Cochrane Risk of Bias Tool. Among 7 RCTs, all the studies reported the methods of random sequence generation but only three studies of two articles employed allocation concealment [31, 36]. Only two studies reported the methods of patient blinding [32, 35], while Zhou et al. [34] admitted that it was at high risk. Methods of blinding of outcome assessment were only applied in two studies [34, 35]. Evaluation of incomplete outcome data depended on whether the clear descriptions of baseline data were shown. According to this standard, all studies were at low risk. None of the studies have published study protocols before, so it was difficult to evaluate selective reporting. We tended to believe a study was at low risk only if it reported all the three secondary outcome measures (FMA, NDS, BI, or MBI). Thus, low risk of bias in the domain of selective reporting was given to four studies [31, 33–35]. In addition, we did not find any other sources of bias. Overall, these studies were not of high quality, mainly in terms of allocation concealment, patient blinding, and outcome blinding.  Figure 2 presents the quality assessment of these 7 included studies.

### 3.3. Meta-Analysis of ReHo Changes

#### 3.3.1. Mean Analysis

**Figure 3:**
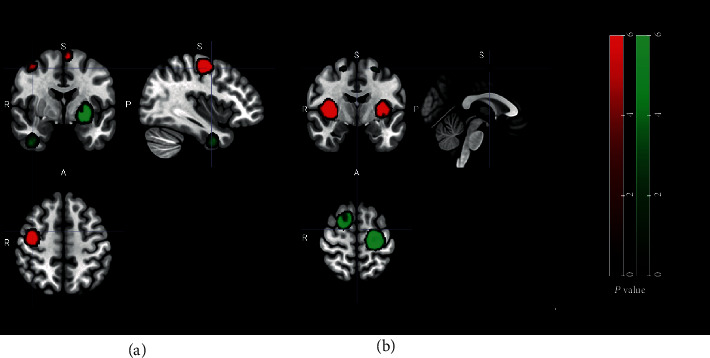
SDM-PSI meta-analysis for (a) ReHo change in poststroke patients after conventional treatment and (b) ReHo change in poststroke patients after acupuncture treatment. Red color refers to hyperactivation, and green color refers to hypoactivation.

Patients in the treatment group showed hyperactivation in the right basal ganglia (*P* < 0.05, *z* = 6.622) and left insula (*P* < 0.05, *z* = 5.232) after acupuncture therapy and the decreased activity of the left precentral gyrus (PG; *P* < 0.05, *z* = −4.477) and right superior frontal gyrus (SFG; *P* < 0.05, *z* = –4.723). Patients in the control group showed hyperactivation in the right middle frontal gyrus (MFG; *P* < 0.05, *z* = 5.457) and left supplementary motor area (SMA; *P* < 0.05, *z* = 4.867) after conventional treatment and a decrease of activity in the left striatum (*P* < 0.05, *z* = –4.876) and left inferior frontal gyrus (IFG; *P* ≤ 0.05, *z* = –5.547). Peak coordinates and cluster breakdowns are shown in Table 2. Differences in the brain regions between conventional treatment and acupuncture treatment have been visualized in Figure 3.

#### 3.3.2. Heterogeneity Analysis and Publication Bias

In the treatment group, the ganglia, insula, left PG, and right SFG showed low between-study heterogeneity of effect size differences in peak coordinates (*I*^2^ = 0.88%–7.40%) and low between-study heterogeneity for each significant peak in the control group (*I*^2^ = 0.09%–1.29%). The heterogeneity results are shown in Table 2. The Egger tests were insignificant in the treatment group (*P* = 0.464) and the control group (*P* = 0.501).

#### 3.3.3. Metaregression Analysis

Metaregression was used to search for potential correlation between FMA scores, mean age, and disease duration in the treatment group. The FMA scores are associated with the left insula (Table S1A). The analysis of mean age shows significance in the brain regions of the left cerebellum (Table S1B). The disease duration is associated with many regions, including the left cerebellum, arcuate network and right SMG, and SFG (Table S1C).

### 3.4. Meta-Analysis of Secondary Outcomes

Compared with conventional treatment, manual acupuncture therapy was associated with a decrease of NDS score (–4.40; 95% CI, 3.79 to 5.01; *I*^2^ = 0), indicating that acupuncture could mitigate the impairment of nervous function. The change in BI score was 3.47 (3.47; 95% CI, 0.99 to 5.95; *I*^2^ = 87%) and that in MBI score was 7.49 (7.49; 95% CI, 5.38 to 9.59; *I*^2^ = 0). The increase of BI or MBI suggested that patients had better daily living ability after acupuncture. A greater increase of FMA score (5.73; 95% CI, 4.35 to 7.11; *I*^2^ = 77%) also demonstrated that acupuncture could improve motor function of the upper and lower limb. The forest plots are shown in Figures S1A–S1D.

## 4. Discussion

### 4.1. Main Findings

It is suggested that acupuncture therapy could relieve neurological deficiency (NDS) and improve motor function (FMA) and daily living ability (MBI). These findings are discussed in many previous meta-analysis studies, so we will not discuss further here [10, 37, 38]. In this paper, we mainly focus on the meta-analysis of ReHo changes. In the treatment group, stroke patients showed hyperactivation in the bilateral basal ganglia and insula. In the control group, patients showed that brain activities were increased in the bilateral frontal gyrus (BA6). However, the decreased activity of the bilateral frontal gyrus (BA6) and left precentral gyrus (BA4) was found in the treatment group. Additionally, the ReHo index of the left insula significantly correlated with FMA scores across all stroke patients after acupuncture therapy.

### 4.2. The Involvement of the Basal Ganglia and Insula in Neuroplasticity Induced by Acupuncture

The results showed that resting-state spontaneous brain activity was obviously increased in the bilateral basal ganglia and insula after acupuncture treatment. Hyperactivation in the basal ganglia comprised mostly the striatum. The basal ganglia play an important integrative role in motor regulation [39]. It is implicated in human dystonia and motor control [40]. Wang et al. have compared ischemic stroke patients to healthy participants, and they found that stroke patients showed low ReHo values in the basal ganglia, which is consistent with previous conclusions that the basal ganglia are vulnerable to ischemia [41, 42]. Regarding motor function, the extent of damage to the basal ganglia seems to be a neural correlate for motor deficit. Three studies included in this review reported that the lesion locations were primarily in the basal ganglia [32, 34, 35], which is in line with the theory that a common ischemic stroke in deep brain regions featured by high disability rate is basal ganglia lacunar stroke [43]. Thus, it can be speculated that limb motor dysfunction of included ischemic stroke patients was mainly related to basal ganglia impairment. The results revealed that acupuncture therapy was also associated with hyperactivation in the insula. The insula integrates different functional systems including sensory motor and cognition and plays a part in motor programing and control [44, 45]. Existing evidence suggests that the anterior insula is commonly affected by acute middle cerebral artery stroke [46]. It seems that the damage of the insula is responsible for the motor dysfunction in stroke patients and acupuncture can activate it to exert therapeutic effect. The metaregression analysis also found that the ReHo increases of the insula are positively correlated to the increase in FMA scores, suggesting that acupuncture could improve ability of daily life via the activation of the insula. Here, our results show an increased spontaneous activity in the basal ganglia and insula after acupuncture treatment, indicating that acupuncture could remodel neuronal function of these areas.

### 4.3. The Involvement of Motor-Related Areas in Neuroplasticity Induced by Acupuncture

In both the treatment and control groups, our results showed significant alterations of brain activation in most parts of motor-related areas, including the bilateral superior frontal gyrus (especially BA6) and left precentral gyrus (especially BA4). BA4 and BA6 are traditional motor areas, consisting of the “classical” motor network. BA4 is the location of the primary motor cortex (M1), responsible for planning and controlling voluntary motor movement on the body's contralateral side. BA6 includes the premotor cortex (PMC) and supplementary motor area (SMA), where neurons have projections to M1 as well as the corticospinal tract (CST) [47]. It bridges prefrontal and primary motor cortices and is engaged in planning of complex and coordinated movements [48, 49]. Evidence from animal and clinical studies suggests that those areas associate with movement preparation and execution and the activation of these areas contributes to reorganization after stroke [50–52]. Therefore, the functional plasticity of BA6 and BA4 might explain the mechanism of motor recovery.

In this meta-analysis, the ReHo value of bilateral BA6 is significantly increased in the control group but decreased in the treatment group. Here, we speculate that the difference between two groups is in connection with the compensatory activity. Compensation at the neuronal level is characterized by activation in alternative brain areas not normally observed in nondisabled individuals [53]. Previous animal studies have demonstrated that reorganization of residual motor cortex contributes to the restoration of movement dysfunction after stroke, which indicates that increased fMRI signal within a given brain area can not only reflect neural recovery but also compensation [54, 55]. Thus, the increased activation of BA6 in the control group should be interpreted as being associated with compensation, which is in accordance with theories that the increased activity of BA6 is a form of compensation for motor deficit after stroke [56]. A longitudinal fMRI study observed, in the early phase of poststroke recovery (less than 6 months), hyperactivation in bilateral SMA and PMC [57]. Another study with similar results identified a positive correlation, involving increased signals in SMA, cingulate, and other motor areas of both hemispheres with good recovery process (from 2 to 7 weeks up to 6 months), suggesting that compensatory network may be the substrates of rehabilitation strategies [58]. All the 7 studies in this review were similar in the duration of symptoms, ranging from 9-14 days up to 6 months, at which status the activation of BA6 would be increased according to the conclusions above. Interestingly, after receiving acupuncture treatment, the activation level of bilateral BA6 is induced to reduce. The decreased activity of BA6 may implicate the reduced dependence on its compensation, indicating the recovery of motor network, thus helping the ipsilateral limb to recover. Similarly, the results revealed that acupuncture therapy was also associated with hypoactivation in left BA4. Evidence from meta-analysis describes that an increased activation in contralesional M1 (BA4) is a highly consistent finding across different impairment levels and times poststroke [59]. This is in accordance with theories that the increased activity of sound side hemisphere is a form of compensation for dysfunction of other damaged cerebral cortex [56]. From this perspective, if most brain lesions of included patients had the right laterality, we would expect to see a weakened compensation of contralesional BA4 by acupuncture. Considering that 4 studies in this review did not report the locations of infarcted lesions, this assumption warrants further experiments and opens a way for investigation of the relationship between acupuncture treatment and compensatory activity of BA4.

### 4.4. Strengths and Limitations

This review is unique and innovative. To our knowledge, we are the first to have SDM-PSI analysis of rs-fMRI used in RCTs of acupuncture. Altered cerebral activity in ischemic stroke patients with motor dysfunction provides a new insight into brain mechanism and clinical application, and this review is intended to serve as both a challenge and an encouragement. Also, strict inclusion criteria were made to minimize selection bias, and 7 studies were synthesized in the final analysis. These trials generally consisted of similar target participants and study designs, thus making results comparable.

As an innovative study, this systematic review still has certain limitations. The main limitation of this review is the small sample size. Only 7 trials and 278 participants met the inclusion criteria so that publication bias and subgroup analysis of secondary outcomes measures were not performed. The results of quality assessments indicated that some studies were of low quality, especially in terms of blinding. The lack of high-quality RCTs made it hard to perform an accurate analysis of the interaction between acupuncture efficacy and neuroplasticity. Thus, to better figure out the potential influence of acupuncture on neuroplasticity after stroke, it is time to organize large-scale and elaborate-designed RCTs for further investigations. Moreover, it is supposed that different acupuncture parameters (referring to different acupoints, duration, frequency, and operation methods) may cause fine distinctions of altered cerebral activity. Therefore, there may exist heterogeneity in the acupuncture therapy protocol between included studies. Considering the particularity of acupuncture treatment, it is significant to synthesize the studies focusing on specific manipulation of acupuncture.

## 5. Conclusion

The meta-analyses of clinical outcome measures (FMA, NDS, BI, or MBI) show that acupuncture treatment is effective for motor function recovery in ischemic stroke patients. The ReHo meta-analysis reveals that acupuncture could induce extensive changes of cerebral activity, which suggests that the alterations of the basal ganglia, insula, and motor-related areas are involved in neuroplasticity of acupuncture. These findings provide a new insight into the mechanisms underlying effectiveness of acupuncture and also lead to the articulation of a more general hypothesis that acupuncture plays a role in facilitating neuroplasticity.

## Figures and Tables

**Table 1:** Clinical characteristics of subjects in the included studies.

Study	Cases (T/C)	Participant					Comparison		Intervention		Outcome measures		
		Condition	Lesion location	Duration (days)	Age (years)	Gender (male/female)	T	C	Acupoints	Regimen	Analysis of fMRI	Coordinate	Secondary outcomes
Zhengfei Ye 2018	84 (42/42)	Poststroke limb motion dysfunction	Unclear	Not reported	T: 55.67 ± 8.38 C: 57.01 ± 6.97	T: 22/20 C: 24/18	Acu+Conv	Conv	LYDDM	7/week for 2 weeks	ReHo	MNI	FMA, BI
Zhihong Chen 2016	60 (30/30)	Poststroke limb motion dysfunction	Unclear	T: 29.83 ± 7.27 C: 30.17 ± 7.55	T: 62.67 ± 3.79 C: 65.50 ± 6.43	T: 17/13 C: 16/14	Acu+Conv	Conv	LYDDM	7/week for 2 weeks	ReHo	MNI	IFMA, BI
Zhihong Chen 2016	60 (30/30)	Poststroke limb motion dysfunction	Unclear	T: 23.33 ± 5.92 C: 30.17 ± 7.55	T: 65.33 ± 5.31 C: 65.50 ± 6.43	T: 18/12 C: 16/14	Acu+Conv	Conv	XNKQ	7/week for 2 weeks	ReHo	MNI	FMA, BI
Nana Chen 2017	12 (6/6)	R limb motion dysfunction	L basal ganglia or corona radiata	T: 14.83 ± 13.848 C: 12.83 ± 14.566	T: 62.00 ± 8.485 C: 67.33 ± 8.779	T: 2/4 C: 4/2	Acu+Conv	Sham acu+Conv	Empirical acupoints	7/week for 2 weeks	ReHo	MNI	FMA, MBI
Ping Wu 2017	21 (11/10)	Poststroke limb motion dysfunction	Unclear	T: 52.818 ± 45.31 C: 52.200 ± 47.852	T: 69.364 ± 12.075 C: 61.300 ± 11.107	T: 7/4 C: 5/5	Acu+Conv	Conv	Empirical acupoints	5/week for 4 weeks	ReHo	Tal	NSD, FMA, MBI
Ximei Xie 2012	20 (10/10)	R limb motion dysfunction	Pons or thalamus or L basal ganglia	T: 19.50 ± 1.66 C: 19.10 ± 1.18	T: 67.70 ± 3.82 C: 64.80 ± 3.72	T: 6/4 C: 5/5	Acu+Conv	Conv	Empirical acupoints	5/week for 4 weeks	ReHo	Tal	NDS, FMA, BI
Yumei Zhou 2015	21 (11/10)	R limb motion dysfunction	L basal ganglia	T: 52.20 ± 34.20 C: 52.80 ± 30.47	T: 69.36 ± 4.06 C: 61.30 ± 7.94	T: 7/4 C: 4/6	Acu+Conv	Conv	Empirical acupoints	5/week for 4 weeks	ReHo	MNI	NSD, FMA, MBI

Abbreviations: Acu: acupuncture treatment; BA: Brodmann area; BI: Barthel index; C: control group; Conv: conventional treatment; FMA: Fugl-Meyer assessment; BMI: modified Barthel index; LYDDM: lifting yang to dredging du meridian needling method; MNI: Montreal Neurological Institute; R: right; ReHo: regional homogeneity; S: sensitivity; SDM: signed differential mapping; T: treatment group; XNKQ: Xingnao Kaiqiao needling method.

**Table 2:** Brain activity changes in patients after treatment compared to baseline.

	MNI coordinates			SDM *z*-score^a^	*P* value^b^	Voxels^c^	Cluster breakdown	*I* ^2^
	*x*	*y*	*z*					
Treatment group								
R basal ganglia	34	-12	8	6.622	<0.05	1244	R lenticular nucleus, putamen, striatum, insula, rolandic operculum, BA48	0.88%
L insula	-32	-14	10	5.232	<0.05	887	L insula, lenticular nucleus, putamen, rolandic operculum, BA48	1.02%
L precentral gyrus	-28	-24	66	-4.477	<0.05	337	L precentral gyrus, corpus callosum, superior frontal gyrus, dorsolateral, BA6	5.83%
R superior frontal gyrus	22	2	62	-4.723	<0.05	20	Right superior frontal gyrus, dorsolateral, BA6	7.40%
Control group								
R middle frontal gyrus	38	-6	56	5.457	<0.05	575	R middle frontal gyrus, superior frontal gyrus, dorsolateral, BA6	0.42%
L supplementary motor area	-6	4	66	4.867	<0.05	83	L supplementary motor area, BA6	1.29%
L basal ganglia	-24	0	-6	-4.876	<0.05	249	L lenticular nucleus, putamen, striatum, insula, BA48	0.09%
L inferior frontal gyrus	-32	32	-14	-5.547	<0.05	166	L inferior frontal gyrus, orbital part, BA47	0.52%

^a^Peak height threshold: *z* > 1. ^b^Voxel probability threshold: *P* < 0.005 uncorrected and remained after correcting threshold (TFCE) of *P* < 0.05. ^c^Cluster extent threshold: number ≥ 10 voxels. Abbreviations: BA: Brodmann area; *I*^2^: heterogeneity *I*^2^; MNI: Montreal Neurological Institute; R: right; ReHo: regional homogeneity; SDM: signed differential mapping.

## Supplementary Materials

Supplementary Materials The supplementary material for this article can be found in the supplementary file. Figure S1A | Forest plot presenting the study meta-analysis for the living quality (nervous deficiency scale). Figure S1B | Forest plot presenting the study meta-analysis for the activity of daily living (Barthel index), Figure S1C | Forest plot presenting the study meta-analysis for the activity of daily living (modified Barthel index), Figure S1D | Forest plot presenting the study meta-analysis for the limb function (Fugl-Meyer assessment), Table S1A Meta-regression analysis of FMA scores in treatment group, Table S1B Meta-regression analysis of age in treatment group. Table S1C Meta-regression analysis of duration in treatment group.
