# Supplementary material for: Effect of Acupuncture on Neuroplasticity of Stroke Patients with Motor Dysfunction: A Meta-Analysis of fMRI Studies
Source: Neural Plast. 2021 Jun 2;2021:8841720. doi: 10.1155/2021/8841720 (PMC8192216; doi:10.1155/2021/8841720)
Supplement: Supplementary Materials — The supplementary material for this article can be found in the supplementary file. Figure S1A | Forest plot presenting the study meta-analysis for the living quality (nervous deficiency scale). Figure S1B | Forest plot presenting the study meta-analysis for the activity of daily living (Barthel index), Figure S1C | Forest plot presenting the study meta-analysis for the activity of daily living (modified Barthel index), Figure S1D | Forest plot presenting the study meta-analysis for the limb function (Fugl-Meyer assessment), Table S1A Meta-regression analysis of FMA scores in treatment group, Table S1B Meta-regression analysis of age in treatment group. Table S1C Meta-regression analysis of duration in treatment group. [file 8841720.f1.docx]

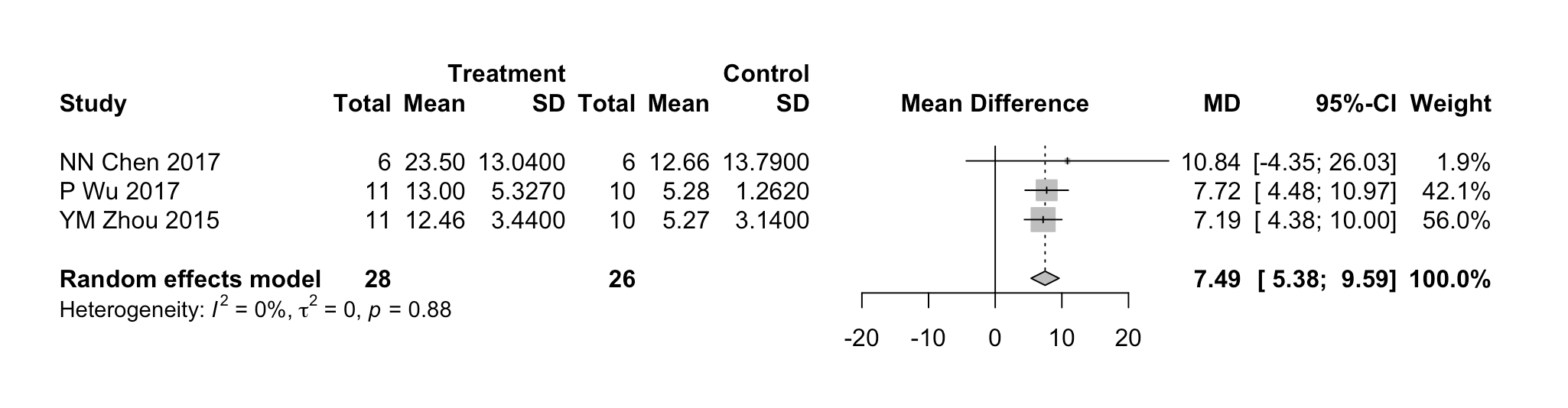


Figure S1A | Forest plot presenting the study meta-analysis for the living quality (nervous deficiency scale


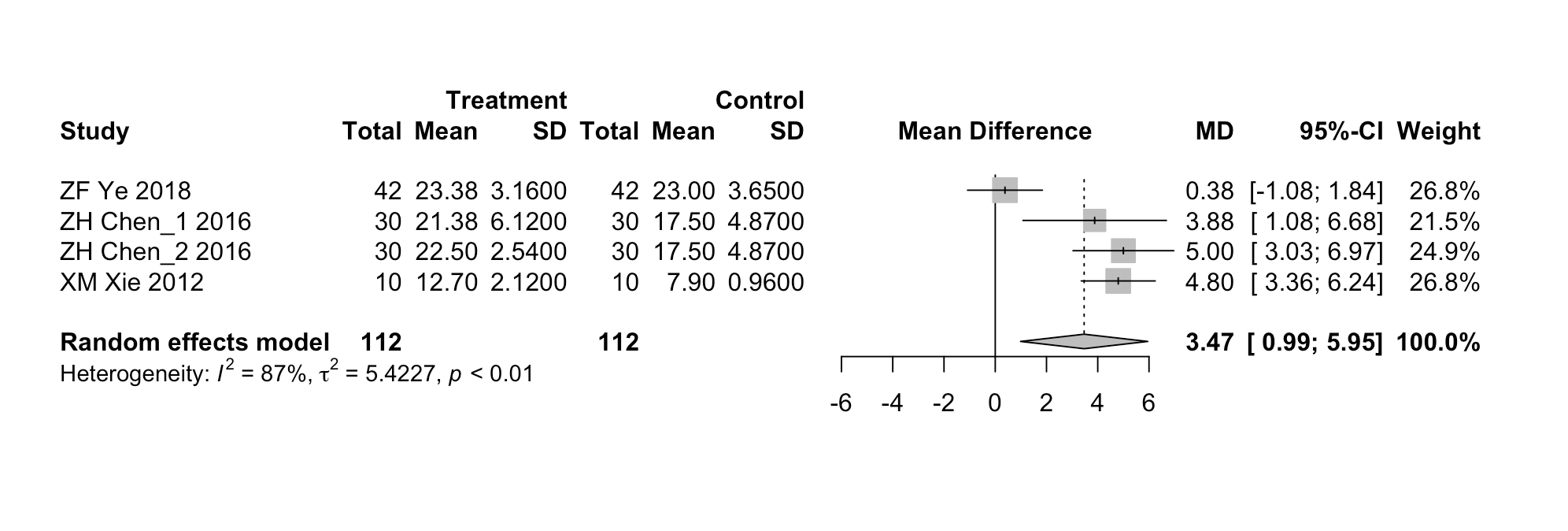


Figure S1B | Forest plot presenting the study meta-analysis for the activity of daily living (Barthel index)

Figure S1C | Forest plot presenting the study meta-analysis for the activity of daily living (modified Barthel index)


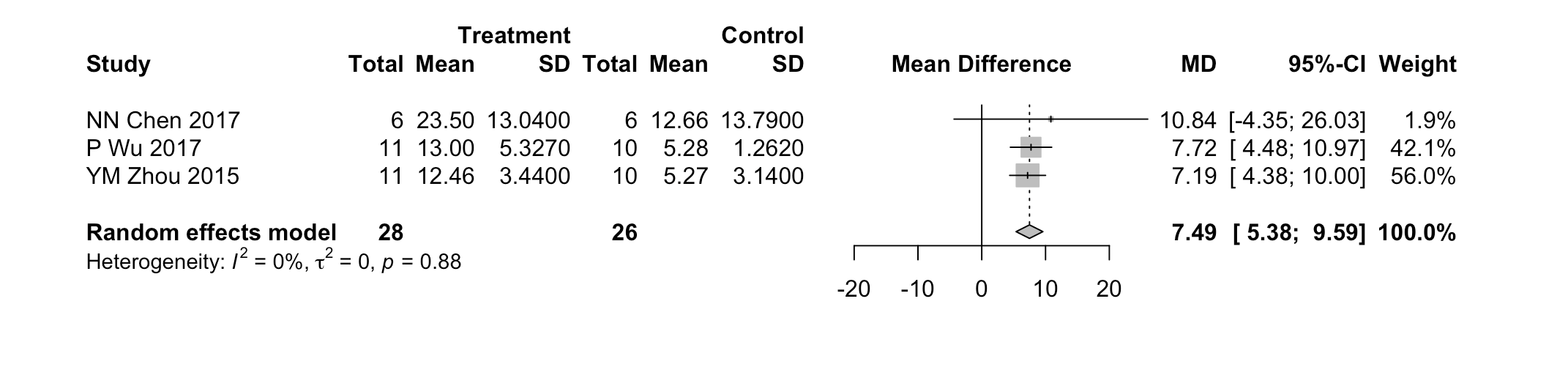


Figure S1D | Forest plot presenting the study meta-analysis for the limb function (Fugl-Meyer assessment)


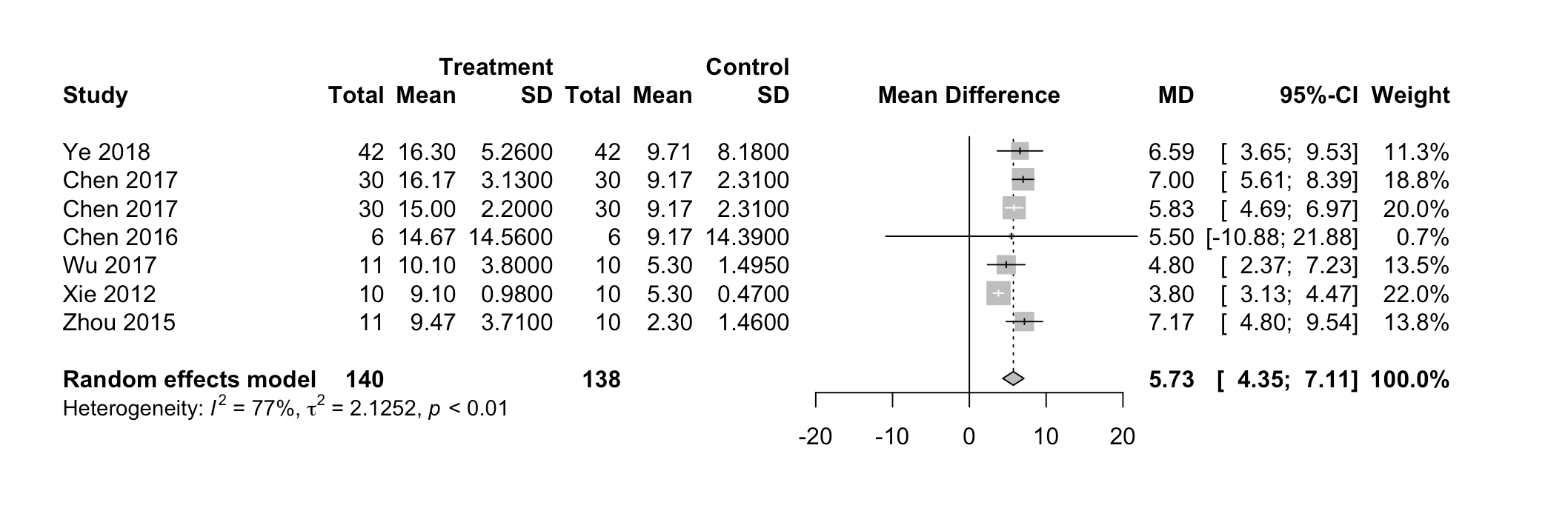


# Table S1A Meta-regression analysis of FMA scores in treatment group

| Region | MNI coordinate | | | SDM z-score ^(a)^ | p-value ^(b)^ | number of voxels ^(c)^ |
| --- | --- | --- | --- | --- | --- | --- |
|  | x | y | z |  |  |  |
| **Effect of FMA scores** |  |  |  |  |  |  |
| L insula | -32 | -2 | 6 | 3.057 | 0.017 | 552 |

^a^Peak height threshold: z > 1;

^b^Voxel probability threshold: p < 0.005;

^c^Cluster extent threshold: number ≥ 10 voxels

Abbreviations: FMA, Fugl-Meyer assessment; L, left; MNI, Montreal Neurological Institute; R, right; SDM, signed differential mapping.

# Table S1B Meta-regression analysis of age in treatment group.

| Region | MNI coordinate | | | SDM z-score ^(a)^ | p-value ^(b)^ | number of voxels ^(c)^ |
| --- | --- | --- | --- | --- | --- | --- |
|  | x | y | z |  |  |  |
| **Effect of age** |  |  |  |  |  |  |
| L cerebellum | -38 | -74 | -42 | 3.675 | 0.003 | 413 |

# Table S1C Meta-regression analysis of duration in treatment group.

| Region | MNI coordinate | | | SDM z-score ^(a)^ | p-value ^(b)^ | number of voxels ^(c)^ |
| --- | --- | --- | --- | --- | --- | --- |
|  | x | y | z |  |  |  |
| **Effect of age** |  |  |  |  |  |  |
| L cerebellum | -38 | -78 | -38 | 3.827 | 0.005 | 616 |
| R supramarginal gyrus | 60 | -44 | 28 | 4.180 | 0.009 | 184 |
| L arcuate network | -44 | -44 | 24 | 4.548 | 0.005 | 152 |
| R superior frontal gyrus | 10 | 40 | 52 | 4.470 | 0.027 | 50 |

^a^Peak height threshold: z > 1;

^b^Voxel probability threshold: p < 0.05;

^c^Cluster extent threshold: number ≥ 10 voxels

Abbreviations: MNI, Montreal Neurological Institute; R, right; SDM, signed differential mapping.
